# Clusters of diet, physical activity, television exposure and sleep habits and their association with adiposity in preschool children: the EDEN mother-child cohort

**DOI:** 10.1186/s12966-020-00927-6

**Published:** 2020-02-12

**Authors:** Cécilia Saldanha-Gomes, Matthieu Marbac, Mohammed Sedki, Maxime Cornet, Sabine Plancoulaine, Marie-Aline Charles, Sandrine Lioret, Patricia Dargent-Molina

**Affiliations:** 1 Université de Paris, CRESS, INSERM, INRA, F-75004 Paris, France; 2 Paris-Saclay University, Faculty of Medicine, F-94276 Kremlin-Bicêtre, France; 3 INSERM, UMR1153 Center of Epidemiology and StatisticS (CRESS), Research Team on Early Life Origins of Health (EARoH), Bat Inserm 15-16, Avenue Paul Vaillant Couturier, 94807 Villejuif Cedex, France; 4 Rennes University, Ensai, CNRS, CREST - UMR 9194, F-35000 Rennes, France; 5 Paris-Saclay University, INSERM UMR1018, CESP, F-94807 Villejuif, France

**Keywords:** Adiposity, Preschoolers, Lifestyle clusters, Latent class analysis, Diet, Physical activity, TV exposure, Sleep

## Abstract

**Background:**

Despite the growing interest in the relation between adiposity in children and different lifestyle clusters, few studies used a longitudinal design to examine a large range of behaviors in various contexts, in particular eating- and sleep-related routines, and few studies have examined these factors in young children. The objectives of this study were to identify clusters of boys and girls based on diet, sleep and activity-related behaviors and their family environment at 2 and 5 years of age, and to assess whether the clusters identified varied across maternal education levels and were associated with body fat at age 5.

**Methods:**

At 2 and 5 years, respectively, 1436 and 1195 parents from the EDEN mother-child cohort completed a questionnaire including behavioral data. A latent class analysis aimed to uncover gender-specific behavioral clusters. Body fat percentage was estimated by anthropometric and bioelectrical impedance measurements. Association between cluster membership and body fat was assessed with mutivariable linear regression models.

**Results:**

At 2 years, two clusters emerged that were essentially characterized by opposite eating habits. At 5 years, TV exposure was the most distinguishing feature, but the numbers and types of clusters differed by gender. An association between cluster membership and body fat was found only in girls at 5 years of age, with girls in the cluster defined by very high TV exposure and unfavorable mealtime habits (despite high outdoor playing and walking time) having the highest body fat. Girls whose mother had low educational attainment were more likely to be in this high-risk cluster. Girls who were on a cluster evolution path corresponding to the highest TV viewing time and the least favorable mealtime habits from 2 to 5 years of age had higher body fat at 5 years.

**Conclusions:**

Efforts to decrease TV time and improve mealtime routines may hold promise for preventing overweight in young children, especially girls growing up in disadvantaged families. These preventive efforts should start as early in life as possible, ideally before the age of two, and should be sustained over the preschool years.

## Background

The overweight and obesity (OW-OB) epidemic spread rapidly in the 1990s and now affects increasingly younger children [1]. This matters because obese children are more likely to become obese adults and experience adverse health consequences [2]. Behavioral factors that directly or indirectly affect the energy balance, such as consumption of energy-dense foods and sodas, high television (including DVD) use, low levels of physical activity (PA) and short sleep time, are considered major drivers of the OW-OB epidemic [3]. These potentially modifiable obesogenic factors tend to co-occur in some individuals and may interact in multiple ways that modify the risk of OW-OB, with potentially synergistic effects [4, 5]. Evermore studies have examined patterns of diet, PA and sedentary behaviors in children and adolescents with exploratory data-driven methods [6–8]. These include cluster analysis, based on either geometric (e.g., k-means) or probabilistic (e.g., mixture models) methods, they are aimed at grouping observations into homogeneous clusters [9]. This approach is attractive in part because of its potential practical value, understanding which obesogenic behaviors need to be targeted together and in whom can provide us with useful information for tailoring interventions to the needs of specific groups at higher risk [6, 10], and thereby increasing the effectiveness of existing pediatric obesity prevention strategies [11, 12].

Reviews of studies using cluster analysis to uncover patterns of diet and/or activity-related behaviors in children and adolescents report that these behaviors cluster in complex ways [6, 8, 13]. Mixed clusters characterized by a mixture of both healthy and unhealthy behaviors being frequently reported (e.g., children who engage in high levels of sports activities but also spend a lot of time on television-based sedentary activities). Cluster membership also differs by gender, age and socio-economic position (SEP) [6]. Some studies describe clusters characterized by combinations of obesogenic behaviors that are associated with higher BMI or higher prevalence of OW-OB, and thus provide support for the hypothesis that specific combinations of behaviors have synergistic effects. Other studies, however, have observed no such association [6]. Nonetheless, most previous studies had cross-sectional designs, only a few used adiposity-specific indicators, and few have considered sleep [14–16], although insufficient sleep duration is recognized as an important risk factor for OW-OB [17] and interactions have been shown with TV use, PA and eating behaviors [4, 18]. Moreover, few cluster studies have included variables capturing contextual information, in particular eating- or sleep-related routines [14, 19], although these data may provide additional useful information for developing targeted interventions. Most studies have been conducted in older children (> 9 years old) and adolescents, and evidence in young children (< 6 years) is scarce [14, 20–22]. Reports that diet, sleep and activity-related habits are formed in the early fast developmental years and have been shown to track into late childhood, adolescence and even adulthood [23], underline the importance of studying the formation and evolution of clusters of behaviors during this period.

The primary aim of the study, using data from the EDEN mother-child cohort, was to identify clusters of children based on diet, sleep and activity-related behaviors and their family environments at 2 and 5 years of age, in boys and girls separately, and to assess whether the clusters identified varied across maternal education levels. Another important goal was to determine if cluster membership at 2 and 5 years was associated with body fat at age 5. Finally, we explored cluster evolution between 2 and 5 years, i.e., whether and how children moved from one cluster to another over this period and examined whether some cluster evolution paths were associated with body fat at 5 years. We hypothesized that children belonging to clusters combining unhealthy behaviors would have a higher percentage of body fat than those belonging to clusters combining healthy behaviors.

## Methods

### Study design and population

The EDEN mother-child study is a prospective cohort designed to assess pre- and postnatal determinants of child health and development [24]. In brief, 2002 pregnant women (< 24-weeks’ gestation) aged 18–44 years were recruited between 2003 and 2006 in two university hospitals located in Nancy and Poitiers, France. Exclusion criteria were multiple pregnancies, history of diabetes, inability to speak or read French and any plan to move out of the region within the next 3 years.

A total of 1903 children were born alive and then followed up periodically by postal questionnaires and clinical examinations. At age 2 and 5, respectively 1436 and 1195 parents (usually the mother) completed a postal questionnaire, the data of which were used to construct the obesity-related behavior clusters of children. At 5 years, 1101 children had a full clinical examination including anthropometric measurements and bioelectrical impedance analysis (BIA).

### Measures

#### Children’s obesity related behaviors

The cluster analysis considered 44 variables (2 continuous) related to the child’s diet, PA, TV use and sleep at age 2 and 40 variables (4 continuous) variables, at age 5. These variables are defined in Table 1. Children’s dietary intake was collected at each of these ages with a food frequency questionnaire. These were short versions of the food frequency questionnaire used by mothers during their pregnancies, which was validated in adults and adolescents [26]. Average time daily spent on TV, outdoor play and walking were assessed from the responses to three questions regarding the time (in min per day) that the child spent watching TV, playing outdoors and walking on a typical weekday (excluding Wednesday), Wednesday (day off school for prekindergarten, kindergarten and elementary schools) and weekend days [25]. The time the child spent playing outdoors was standardized by season to take seasonal variations in outdoor PA into account. Night sleep duration was calculated based on parent’s reports of bedtime and wake up time [27]. At age 2, total sleep duration included night sleep and nap durations.

**Table 1:** Variables considered for clustering at age 2 and 5 in each behavioral domain

Input variables	Categories/units
Diet	
27 food groups from FFQ: Dairy yogurt and cottage cheese, dairy puddings/ice cream, cheese, rice/pasta/semolina, potatoes, French fries (chips), pizza/pie, legumes, cooked vegetables, raw vegetables, ham/poultry, red meat, processed meat, high fat fish, low fat fish, eggs, fresh fruit, stewed fruit, fruit juice, soft drinks, diet soft drinks, breakfast cereals, bread, biscuits (cookies), chocolate/candy, potato chips (crisps), milk (only at age 5)	Never, < 1 time/month, 1–3 times/month, 1–3 times/week, 4–6 times/week, 1 time/day, several times/day
Milk intake (only at age 2)	Never, 1 bottle/day, 1–2 bottles/day, 2–3 bottles/day, > 3 bottles/day
Sweetened beverages usually consumed at mealtimes	yes/no
Breakfast daily intake	yes/no (age 2) or never, sometimes, often, always (age 5)
Ready-prepared baby foods consumers^a^ (only at age 2)	yes/no
Snacking outside meals (only at age 5)	≥ 2 times/day, 1 time/day, 2–3 day/week, less often, never
Television exposure	
TV and DVD watching time^b^	0 min/day, > 0 min/day- ≤ 30 min/day, > 30 min/day- ≤ 1 h/day, > 1 h/day (age 2) or continuous variable in min/day (age 5)
Having TV on during meals	never, sometimes, often, always
Physical activity	
Outdoor play time^b^ (standardized by season^c^)	Continuous variable in min/day
Means of transport for walks (only at age 2)	‘on foot’, ‘in a stroller’, ‘about the same on foot and in a stroller’
Regular swimming pool attendance	yes/no (age 2) or never, < 1 time/month, 1 time/month, 2–3 times/month, ≥1 time/month (age 5)
Frequency of physical activities done with mother/father (only at age 2): Going out to walk with father/mother, Playing ball with father/mother	Never, < 1 time/week, 1–2 times/week, 3–5 times/week, daily
Walking time^b^ (only at age 5)	Continuous variable in min/day
Participation in organized sportive activity (only at age 5)	yes/no
Sleep	
Sleep duration^d^	Continuous variable in hours/day
Difficulties falling asleep	‘every evening’, ‘often’, ‘every other night’, ‘sometimes’, ‘never’
Regular bedtime (only at age 2)	yes/no
Regular wake-up time (only at age 2)	yes/no
Intake of bottle of milk at bedtime (only at age 2)	yes/no

Data were managed similarly at 2 and 5 years of age and for outdoor play time and walking time

*Abbreviations*: *FFQ* Food frequency questionnaire, *min* minute

^a^consumption of baby-specific foods sold commercially

^b^assessed from the responses to three questions depending on the day of the week, described elsewhere in detail [25]

^c^to take seasonal variations in outdoor physical activity into account

^d^Total sleep duration (night and nap) at age 2 and night sleep duration at age 5

#### Children’s anthropometric and BIA measurements

Anthropometric and BIA data were collected during the clinical examination that took place at the Nancy and Poitiers university hospitals when the child was 5 years old (mean 5.6 years, standard deviation 0.2 years). Trained investigators using standard procedures performed measurements. The children’s heights were measured as they stood barefoot, their weights while they wore light underwear and skinfold thickness at the left triceps and subscapular sites, with a Holtain skinfold caliper. Height and weight were measured twice and skinfold thickness three times. Children underwent BIA measurements twice, with a single-frequency impedance analyzer (Model BIA 101, Akern-RJL, Italy) after 5 min of rest and with an empty bladder [25]. Body fat percentage was estimated from the mean of each anthropometric and BIA measurements and calculated with the equation of Goran et al. [28] BMI at 2 and 5 years was estimated from model-based individual weight and height trajectories, as described in detail elsewhere [29], which allowed us to calculate the prevalence of overweight, including obesity at 2 and 5 years from the International Obesity Task Force cut-offs [30].

#### Covariates

Maternal education, measured at inclusion in the cohort, was used as a proxy of SEP and defined by the highest diploma obtained: less than high-school, high school diploma, 2-year university degree and ⩾ 3-year university degree). BMI estimated at age 2 [29] was used in longitudinal analyses as a proxy for baseline body fat.

### Statistical analyses

Descriptive statistics were first calculated for maternal and child characteristics at 2 and 5 years. Chi square tests were used to examine gender differences for categorical and t-tests for continuous variables.

**Fig. 1:**
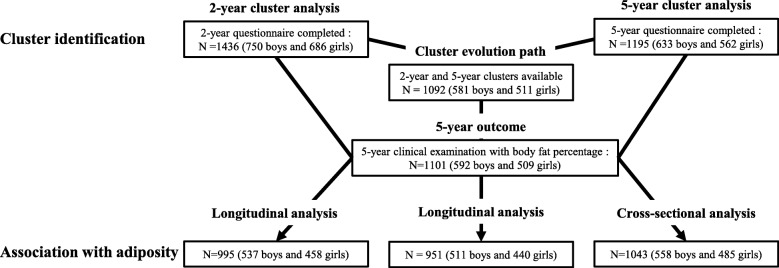
Study samples for the analyses

Finite mixture models, separate for boys and girls, were used to identify the clusters of children [9, 31]. This latent class analysis method enables the management of mixed types of data (i.e. categorical and continuous) without preliminary data conversion by modeling the distribution of the observed variables with a mixture of parametric distributions and an assumption of their independence within clusters. Clusters are then defined as the subset of children drawn by the same mixture component. The method provides not only a partition but also the posterior class membership probabilities for each individual (based on model parameters and the individual’s observed values). Simultaneously, the finite mixture model permits the selection of variables for clustering [32, 33]. Using Marbac and Sedki’s approach [33], implemented in the R package VarSellCM [32], we can perform full model selection, that is, detect the relevant variables for clustering and selection of the number of clusters, according to the Bayesian information criterion (BIC) [34]. By assuming that only a subset of the variables explains the partition, the selection of variables facilitates interpretation of the results and increases the accuracy of the estimators. In a clustering context, a variable is said to be irrelevant for clustering when its distribution is the same in each of the mixture components. The relevance of a given variable for the clustering is thus measured by its discriminating power (see [32] equation~ 3), which makes it possible to sort the variables according to their influence on the construction of the clusters. Models were selected by considering a maximum of seven clusters and assuming that each variable might or might not be relevant to the clustering. For a fixed number of clusters, variable selection according to the BIC and maximum likelihood inference are simultaneously performed by a specific Expectation-Maximization algorithm that deals with missing values by assuming that they are missing completely at random. The analysis thus included the full sample of children at each age, i.e., 1436 2-year children (including 489 with one or more missing behavioral data) and 1195 5-year children (including 330 with one or more missing behavioral data) (see Fig. 1). Each child was assigned to the cluster to which he or she had the highest estimated probability of belonging (Maximum A Posteriori rule). Clusters were named according to their most distinctive characteristics (considering only variables with a discriminating power of at least 5%). Chi square tests were used to examine the association between cluster membership and maternal education level.

To examine how clusters of children evolved from the ages of 2 to 5 years, we cross-classified them according to their cluster membership at both ages and present the proportion of children from cluster at age 2 that moved to each cluster at age 5. Based on the cross-classification, each child was assigned to a given cluster evolution path from 2 to 5 years of age.

Gender-stratified multivariable linear regression models were then used to assess the association between body fat percentage at 5 years and cluster membership at age 2 and at age 5 as well as the cluster evolution path. The reference in each case was the most favorable (a priori) cluster/evolution path. These analyses were conducted in two steps. Model 1 was adjusted for study center, exact age at the 5-year clinical examination, and predicted BMI at age 2 (longitudinal and cluster evolution path analyses only). Model 2 was further adjusted for maternal education. Figure 1 presents the number of children included in each of these regression analyses. SAS 9.3 was used for the multivariate regression analyses. The level of significance set was at *P* < 0.05.

## Results

**Fig. 2:**
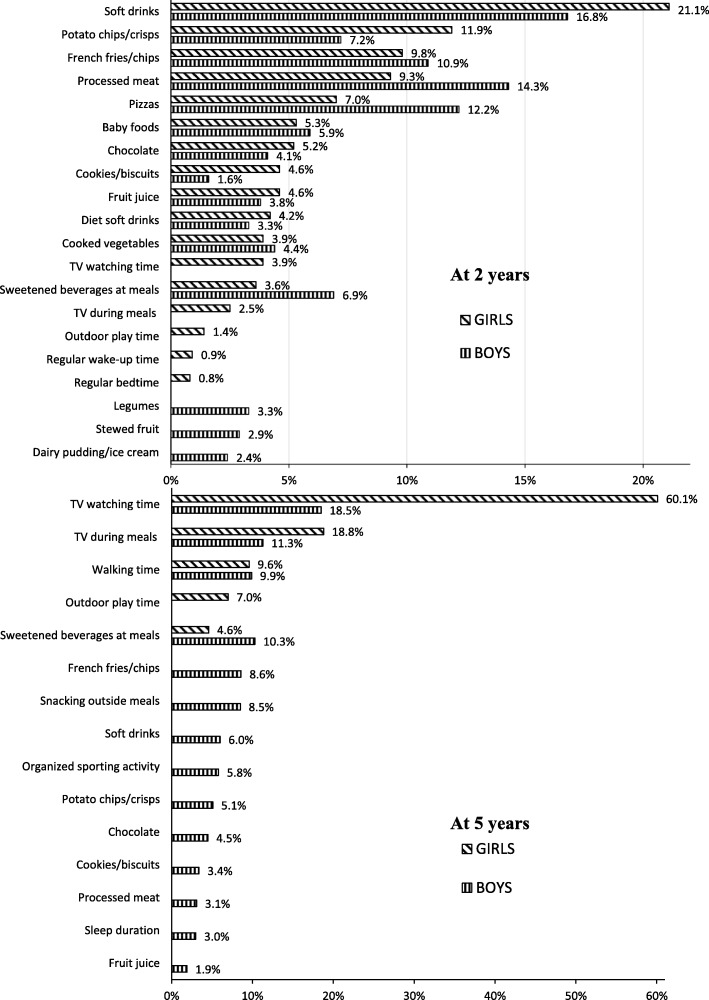
Percentage of discriminating power of the relevant variables for clustering by gender at 2 and 5 years of age

Table 2 shows characteristics of mothers and children at birth, 2 and 5 years of age. Additional Table 1 summarizes the BIC and number of relevant variables for each gender- and age-specific model considered in the cluster analysis, while Fig. 2 shows the relevant variables for the best-fit models, along with their discriminating power. Interpretation of the clusters was based on the probabilities of responses (categorical variables) and means and standard deviation (continuous variables) of the relevant variables within each cluster, reported in Additional Tables 2 to 5.

**Table 2:** Population characteristics by gender (values are percentages unless stated otherwise). The EDEN mother-child cohort

		Boys	Girls
Maternal characteristics at inclusion or delivery		*N* = 750	*N* = 686
Maternal education	No diploma	24.1	23.6
	High school diploma	16.9	18.7
	2-year university degree	23.1	22.3
	≥ 3-year university degree	35.9	35.4
Maternal age at delivery (y), mean (SD)		29.8 (4.7)	29.8 (4.8)
Primiparous (yes)		46.6	48.3
Child behavioral characteristics at 2 years of age		*N* = 750^b^	*N* = 686^b^
French fries/chips	Never	8.7	8.7
	< 1 time/month	18.8	19.4
	1–3 times/month	47.4	44.3
	1–3 times/week	23.4	26.0
	4–6 times/week	1.5	1.3
	1 time/day	0.1	0.3
	Several times/day	0.1	0.0
Sweetened beverages at meals (yes)		**20.5**	**12.4**
Cooked vegetables	Never	1.2	0.7
	< 1 time/month	1.5	1.0
	1–3 times/month	7.3	6.6
	1–3 times/week	22.8	22.8
	4–6 times/week	26.8	26.1
	1 time/day	29.0	29.0
	Several times/day	11.4	13.8
TV/DVD watching time	0 min/day	12.3	11.8
	> 0 to ≤30 min/day	40.5	43.8
	> 30 to ≤60 min/day	25.9	21.4
	> 60 min/day	21.3	23.0
TV on during meals	Never	39.3	41.2
	Sometimes	26.5	28.6
	Often	21.1	19.4
	Always	13.2	10.8
Total sleep duration, h/d, mean (SD)		13 h09 (0 h58)	13 h13 (0 h59)
Outdoor play time, h/d, mean (SD)	Spring	**2 h11 (1 h07)**	**1 h55 (1 h08)**
	Summer	2 h37 (1 h19)	2 h31 (1 h09)
	Autumn	**1 h50 (1 h05)**	**1 h37 (0 h57)**
	Winter	1 h17 (0 h46)	1 h09 (0 h42)
Child anthropometry at 2 years of age		*N* = 747	*N* = 686
Prevalence of overweight (including obesity), IOTF ^a^		4.8	4.1
Child behavioral characteristics at 5 years of age		*N* = 633^b^	*N* = 562^b^
French fries/chips	Never	0.5	0.5
	< 1 time/month	12.0	11.4
	1–3 times/month	51.4	56.2
	1–3 times/week	34.6	30.6
	4–6 times/week	1.1	0.7
	1 time/day	0.5	0.4
	Several times/day	0.0	0.2
Sweetened beverages at meals (yes)		19.8	16.6
Cooked vegetables	Never	0.8	0.5
	< 1 time/month	2.5	1.1
	1–3 times/month	7.6	7.5
	1–3 times/week	32.2	33.9
	4–6 times/week	29.5	28.4
	1 time/day	20.8	18.9
	Several times/day	6.7	9.6
TV/DVD watching time, h/d, mean (SD)		**1 h23 (0 h47)**	**1 h15 (0 h47)**
TV on during meals	Never	35.8	36.5
	Sometimes	32.4	34.9
	Often	21.2	19.6
	Always	10.6	9.1
Participation in organized sports (yes)		**48.2**	**51.8**
Outdoor play time, h/d, mean (SD)	Spring	1 h53 (0 h56)	1 h44 (0 h50)
	Summer	1 h59 (1 h07)	1 h50 (0 h56)
	Autumn	1 h24 (0 h44)	1 h16 (0 h46)
	Winter	**1 h24 (1 h00)**	**1 h04 (0 h46)**
Night sleep duration, h/d, mean (SD)		10 h51 (0 h29)	10 h54 (0 h27)
Child anthropometry at 5 years of age		*N* = 592	*N* = 509
Prevalence of overweight (including obesity) IOTF ^a^		**5.7**	**9.4**
Body fat percentage, mean (SD)		**12.5 (2.8)**	**16.6 (3.2)**

Bold figures indicate statistical significance (*p* < 0.05) for Chi^2^ test or t-test

*Abbreviations*: *h* hours, *d* day, *SD* Standard deviation

^a^International Obesity Task Force cut-off

^b^N may deviate from total sample size because of missing values for behavioral variables

At age 2, the selected model contains two clusters with 15 relevant variables for boys and 17 for girls. In both genders, the most discriminant variables corresponded to intake of energy-dense nutrient-poor (EDNP) food items such as soft drinks and processed and fast foods (e.g., processed meat, pizzas, French fries and potato chips). Because the two clusters were essentially characterized by opposite eating habits, cluster 1 was labeled ‘unhealthy eating’ and cluster 2 ‘healthy eating’. Among girls, the two clusters also had contrasting TV exposure (TV watching time and TV on during meals), PA (playing outdoor) and sleeping habits, with low TV/PA and regular sleeping routines clustered positively with healthy eating habits. The probability of belonging to the assigned cluster exceeded 80% for 88% of the boys and 80% of the girls. Children whose mothers had a lower educational level were more likely to belong to the ‘unhealthy eating’ cluster (*p* value < 10^− 4^).

At age 5, the selected model contains 2 clusters and 14 relevant variables for the boys, and 4 clusters and only 5 relevant variables for the girls. In both genders, TV exposure variables were the most discriminant. In boys, the two clusters differed mainly regarding their TV exposure and eating habits (intake of EDNP food, snacking, soft drinks at mealtimes), with high TV exposure clustered positively with unhealthy eating habits (cluster 1 was therefore labeled ‘high TV–unhealthy eating’ and cluster 2 labeled ‘moderate TV–healthy eating’). The two clusters also differed regarding types of PA: boys in the ‘high TV–unhealthy eating’ cluster spent more time walking, while boys in the ‘moderate TV–healthy eating’ cluster were more likely to participate in organized sports activities. Membership probabilities were greater than 80% for 85% of boys. Boys whose mothers had a lower educational level were more likely to belong to the ‘high TV– unhealthy eating’ cluster (*p* value < 10^− 4^). In girls, the four clusters that emerged were mainly characterized by different activity (TV/PA) patterns, with TV viewing time being by far the most discriminant variable (mean TV time across clusters ranged from 35 to 174 min). The clustering of TV exposure and PA (outdoor playing/walking) was complex, with all possible combinations of favorable and unfavorable behaviors observed; cluster 1 was labeled ‘low TV–low outdoor PA’, cluster 2 ‘moderate TV–rather high outdoor PA’, cluster 3 ‘high TV–low outdoor PA’ and cluster 4 ‘very high TV–high outdoor PA’. TV during meals and sweetened beverages at mealtimes clustered positively with overall TV time. Membership probabilities exceeded 80% for more than 60% of the girls assigned to clusters 1 to 3 and for more than 80% of those assigned to cluster 4. Girls whose mothers had a low educational level were more likely to belong to the ‘very high TV–high outdoor PA’ cluster whereas girls with more highly educated mothers were more likely to belong to the ‘low TV–low outdoor PA’ cluster (*p* value < 10^− 4^).

**Fig. 3:**
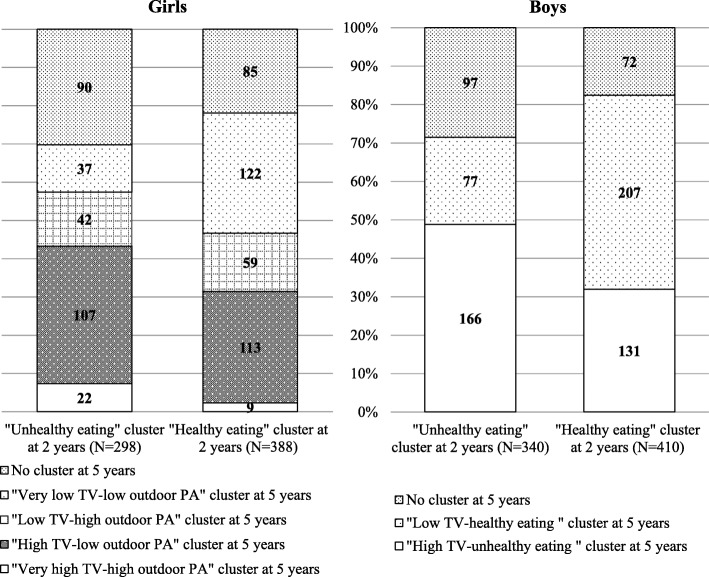
Distribution of children in clusters at 5 years among clusters at 2 years by gender. PA: Physical Activity

Figure 3 shows how children evolved from each cluster at age 2 into clusters at age 5. In both genders, a higher proportion of mothers of children from the ‘unhealthy eating’ versus the ‘healthy eating’ age 2 cluster did not complete the 5-year-questionnaire (29% vs. 20%; Chi^2^ test *p* value ≤10^− 4^). The mothers who did not respond had a higher rate of ‘no diploma’ (31% versus 22%, Chi^2^ global test *p* value = 0.0005). Although the clusters differed at age 2 and age 5, children who belonged to the ‘unhealthy eating’ age 2 cluster were more likely to move to the age 5 clusters characterized by unhealthy eating habits and/or higher TV exposure. There was also a relatively high cross-over between predominantly favorable and unfavorable (based on eating habits and TV) clusters. For example, of the girls in the ‘healthy eating’ cluster at age 2, the same proportions moved to the age 5 clusters with high and with low TV exposure.

The associations between cluster membership and body fat percentage at 5 years are presented in Table 3. The clusters at age 2 were not significantly associated with body fat percentage at 5 years for either gender. Cross-sectional analysis at 5 years showed a significant association between cluster membership and body fat percentage in girls only, with a trend towards increasing body fat percentage that increase with increasing TV exposure across clusters. With the ‘moderate TV–rather high outdoor PA’ cluster as the reference, girls belonging to the ‘very high TV–high outdoor PA’ cluster had significantly higher body fat percentage, even after adjustment for maternal education level (+ 1.53%). Examination of the evolution of the clusters from 2 to 5 years of age showed that, compared with the girls who moved from the ‘healthy eating habits’ cluster at age 2 to the ‘moderate TV–rather high outdoor PA’ cluster at age 5 (reference group), those who moved from the ‘unhealthy eating habits’ cluster at age 2 to the ‘very high TV–high outdoor PA’ cluster at age 5 had a significantly higher body fat percentage (+ 1.82%) at age 5 for a given BMI at age 2, and even after adjustment for maternal education level. There was no significant association between the cluster evolution path and body fat percentage in boys.

**Table 3:** Association between cluster membership at 2 and 5 years of age and cluster evolution path (from 2 to 5 years of age) with body fat percentage at 5 years, by gender

	N	Body fat percentage	
		Linear regression beta coefficients (95% CI)	
		Model 1^a^	Model 2^b^
Clusters at 2 years of age			
Boys			
‘Unhealthy eating’	226	−0.05 (−0.48 to 0.38)	−0.13 (− 0.56 to 0.30)
‘Healthy eating’	311	ref	ref
Girls			
‘Unhealthy eating’	192	0.40 (−0.12 to 0.92)	0.19 (− 0.35 to 0.74)
‘Healthy eating’	266	ref	ref
Clusters at 5 years of age			
Boys			
‘High TV–unhealthy eating’	297	0.23 (−0.24 to 0.70)	0.13 (−0.36 to 0.62)
‘Moderate TV–healthy eating’	261	ref	ref
Girls			
‘Very high TV–high outdoor PA’	33	**1.70 (0.46 to 2.93)**	**1.53 (0.28 to 2.78)**
‘High TV–low outdoor PA’	210	0.23 (−0.53 to 0.99)	0.27 (−0.49 to 1.04)
‘Moderate TV–rather high outdoor PA’	96	ref	ref
‘Low TV–low outdoor PA’	146	−0.41 (−1.22 to 0.40)	− 0.20 (−1.03 to 0.64)
Evolution of clusters			
Boys			
Unhealthy eating at 2 y to high TV at 5 y	148	0.36 (−0.19 to 0.91)	0.18 (−0.39 to 0.75)
Unhealthy eating at 2 y to moderate TV at 5 y	64	0.03 (−0.69 to 0.75)	−0.04 (− 0.76 to 0.68)
Healthy eating at 2 y to high TV at 5 y	122	**0.61 (0.03 to 1.19)**	0.46 (−0.14 to 1.05)
Healthy eating at 2 y to moderate TV at 5 y	177	ref	ref
Girls			
Unhealthy eating at 2 y to very high TV at 5 y	20	**2.03 (0.60 to 3.46)**	**1.82 (0.37 to 3.28)**
Unhealthy eating at 2 y to high TV at 5 y	92	0.42 (−0.53 to 1.37)	0.37 (− 0.59 to 1.32)
Unhealthy eating at 2 y to moderate TV at 5 y	37	−0.22 (−1.40 to 0.95)	− 0.35 (− 1.53 to 0.84)
Unhealthy eating at 2 y to -low TV at 5 y	33	− 0.30 (− 1.51 to 0.91)	−0.23 (− 1.44 to 0.98)
Healthy eating at 2 y to very high TV at 5 y	8	1.17 (−0.88 to 3.22)	1.13 (− 0.92 to 3.18)
Healthy eating at 2 y to high TV at 5 y	98	0.17 (−0.77 to 1.11)	0.23 (−0.71 to 1.17)
Healthy eating at 2 y to moderate TV at 5 y	50	Ref	Ref
Healthy eating at 2 y to low TV at 5 y	102	−0.38 (−1.31 to 0.55)	− 0.19 (− 1.13 to 0.76)

Bold figures indicate 95% CI not including 0

*Abbreviations*: *CI* Confidence interval, *PA* Physical activity, *y* Years of age

^a^ model 1 is a linear model adjusted for study center (Poitiers or Nancy) and exact age at the 5-year clinical examination and predicted BMI at age 2 (only for clusters at age 2 and cluster evolution)

^b^ model 2 is a linear model adjusted as for model 1 and further adjusted for maternal education

## Discussion

Using latent class analysis, we identified distinct clusters of children based on patterns of diet, PA, TV use, and sleep that differed at 2 and 5 years, and were gender-differentiated by the age of 2. Clusters defined by unhealthy diet and/or higher TV exposure were consistently characterized by unfavorable mealtime habits and associated with low maternal educational level. We found an association between cluster membership and body fat percentage only in girls at 5 years of age, this percentage was highest among those in the ‘very high TV–high outdoor PA’ cluster. Furthermore, girls who were on a cluster evolution path corresponding to the highest TV viewing time and the least favorable mealtime habits from 2 to 5 years of age had a higher body fat percentage at 5 years.

Few studies have aimed to identify clusters of young children (< 6 years) based on their patterns of diet, PA, sedentary behavior and sleep [14, 20–22]; none of them concurrently examined behaviors from all four domains and they captured only limited contextual information. Three literature reviews have analyzed the results of studies using cluster analysis to identify lifestyle patterns, in particular diet and/or activity-related behaviors, in school-aged children and adolescents [6, 8, 13]. Despite differences in population sample characteristics, the range and contexts of behaviors considered, and the behavior measurement tools and clustering methods used, some results have been consistent and remain so in our study. In particular, we found that a large number of young children fell into mixed activity (sedentary behavior/PA) clusters, here characterized by high TV and high outdoor PA (playing and/or walking) or vice versa. Among girls, such mixed TV/outdoor PA (playing) patterns emerged as early as 2 years and continued at 5 years, along with healthy and unhealthy activity patterns. Also consistent with previous studies [6], we found that clusters defined by poor diet were common in boys. Among the 5-year-old boys in our study, higher EDNP food/drink intake clustered positively with higher TV exposure, a finding in agreement with the results of a recent study reporting a cluster of ‘energy-dense consumers who watch TV’ that is more likely to include boys among a small sample of 5- to 6-year-old Australian children [22]. Furthermore, our finding that poor eating habits co-occur with high TV exposure echoes the ‘TV-snacking’ pattern consistently reported in studies using principal component analysis (PCA) to examine behavior correlation patterns in samples of children and adolescents around the world [7, 35–37]. Boys score higher than girls on this unhealthy pattern [7].

An especially original aspect of this study is that it collected and considered a large range of variables capturing the context in which obesity-related behaviors occur, especially eating- and sleep-related habits rarely taken into account in previous studies [14, 19, 38]. In particular, we found that variables describing the mealtime setting, i.e., eating with the TV on and consuming sweetened beverages with meals, were useful for distinguishing groups of children with different levels of TV exposure and/or different EDNP food/drink intakes, and discriminated the children with lower TV viewing time and EDNP food/drink intake especially well. In a large cross-sectional study of patterns of sedentary and exercise behaviors among 11-year-old children in nine European countries, te Velde et al. [19], also found that daily dining in front of the TV set discriminated the “healthy” cluster (low sedentary behavior and high physical exercise) quite well. These findings are concordant with the results of a study of 5-year-old Dutch children [38], reporting that children who scored high on the ‘Television-Snacking’ pattern often ate with the TV on and were more likely not to eat at the table. Furthermore, we found that eating with the TV on and consuming sweetened beverages at mealtimes consistently clustered together and that clusters characterized by such unfavorable mealtime habits and higher overall TV exposure comprised proportionally more children growing up in lower SEP families. These results support the idea that family routines and social interactions around mealtimes have major influence in shaping children’s eating and TV-related behaviors from a very early age; they strongly suggest that the mealtime setting might be an important target for family-based interventions, especially those targeting lower SEP families. We also note that sleep-related variables had a low discriminant power in our sample, although regular bedtime and wake-up times and long sleep durations clustered, as expected, with lower TV exposure and healthy eating habits [18, 39].

Finally, we examined the cross-sectional and longitudinal associations between cluster membership and body fat percentage. A significant cross-sectional association was observed between cluster membership and body fat at age 5 among girls: compared with the reference cluster characterized by moderate TV watching time (1 h/day on average) and rather high outdoor PA, girls in the cluster with very high TV watching time (3 h/day on average) and high outdoor PA had significantly higher body fat percentages around 10% higher than the average of 16.6% in the sample of girls. Body fat percentage appears to increase across clusters with increasing average TV viewing time within clusters, regardless of outdoor PA (playing/walking). In particular, the clusters at each end of the body fat percentage distribution were the two mixed TV/PA clusters. These two ‘extreme’ clusters differed markedly for maternal educational level; about half of the girls in the ‘very high TV–high outdoor PA’ cluster had a mother with no diploma. This equivocal finding may be explained by preschoolers’ need for encouragement and support from their parents or care givers to be physically active and play energetically, and this might be even more true for girls [40–43]. There is also evidence that children whose mothers have a lower educational level tend to receive less support and encouragement for PA than children whose mothers are more highly educated [44]. Hence, despite spending a lot of time outdoor, girls in the ‘very high TV–high outdoor PA’ cluster may not accumulate sufficient PA, especially moderate-vigorous PA, to compensate for clearly excessive TV time. The tendency we have observed for the body fat percentage to increase with TV exposure also supports the results of studies among preschool-age children, showing that children who watch more TV tend to accumulate less overall (accelerometer-assessed) PA than children who watch less TV; with both light and moderate-vigorous PA are lower among those with greater TV exposure [45–47]. Examination of cluster evolution paths further suggests that girls who were on a behavioral path characterized by continued high TV exposure and an unfavorable mealtime setting from age 2 had a higher body fat percentage at age 5. Our results further tend to show that girls in low SEP families are more likely to start along this obesogenic behavioral path very early. Longitudinal research in larger samples and over longer time frames are important to confirm these findings.

We found no significant association between cluster membership or evolution path and body fat percentage in boys. This may be partly explained by the fact that boys are generally more active than girls and tend to spend more time in active play and moderate-vigorous PA in general [48, 49]. In addition, adiposity rebound is physiologically later in boys than girls, and 5-year old boys have significantly less body fat than girls [50], as our study confirms. Hence, we cannot rule out the possibility that a small but cumulative effect of poor diet and higher TV exposure on body fat accrual might become more apparent at a later age. Although the number of longitudinal studies is small, those among preschoolers have shown that an unhealthy pattern characterized by more TV time, more snacking and higher consumption of EDNP food/drinks is associated with higher BMI [22, 36].

A limitation of this study includes a reliance on parental report for all behavioral data, it is known to be vulnerable to both recall and social desirability biases. Furthermore, although PA and sedentary behavior take place in multiple contexts, we measured only a selection of children’s possible activities throughout the day. This prevents us from having a complete profile of their activity-related behavior. This may be particularly problematic for girls who tend to spend large amounts of time in non-TV sedentary activities that are seldom measured [44]. Combining activity data collected via accelerometry (thus, objective measures of time spent in activities at different levels of intensity) and via time use diaries (to capture valuable contextual information) would be useful for confirming and building upon the findings presented here. It is, however, difficult to collect these types of data in large epidemiological studies. Another limitation is related to attrition in the EDEN cohort, which, as generally observed in this type of epidemiological study, is proportionately higher for children of low SEP families. Prevalence of overweight and obesity at 5 years was also lower than in the general population, that is, 7.7% vs 11.9–13.5% [51]. This may partly explain the relatively small size of the ‘very high TV–high outdoor PA’ cluster of 5-year-old girls. Furthermore, given that the initial EDEN population of children has a relatively higher SEP than the general population of children of same age, the number of high-risk girls with this particular unhealthy activity-related pattern in the overall population is probably underestimated. Replicating our findings in larger and more socially diverse samples of young children appears important.

Despite these limitations, the study has several strengths including its longitudinal design, the young age of the children, the large range and types of behaviors considered, the use of an adiposity-specific outcome measure and the gender-stratified analyses. Furthermore, our analytical approach is based on latent class models, which involve fewer subjective decisions than other clustering techniques do and presents several advantages, specially dealing with missing values and mixed-type variables, as well as the possibility of full model selection. Another major advantage of our method is that it provides cluster membership probabilities for each child, which is an effective way of assessing the robustness of the classification.

## Conclusions

In summary, findings of this study suggest that there are gender differences in the clustering of diet, outdoor PA, TV exposure and sleep as well as in the various contexts of these behaviors that emerge very early in life and are reinforced as children age. We also found some evidence of a cumulative effect of unhealthy eating habits and continued high TV exposure from the age of two onward, on later body fat in girls; this result supports an integrated approach to obesity prevention. In particular, our findings suggest that efforts to decrease TV time and improve mealtime habits may hold particular promise for the prevention of OW-OB in young children, especially girls from low SEP families. Our findings indicate that these preventive efforts should start as early in life as possible, ideally before the age of two, and should be sustained over the preschool years. More quantitative and qualitative studies are needed to understand the social and cultural settings from which specific obesity-inducing behavior clusters emerge and by which mechanisms and, thereby inform the content of targeted, adapted and gender-sensitive interventions. Studies aimed at developing a more in-depth understanding of the complex interplay between familial and environmental factors in influencing activity-related behaviors and of the early gendered differences in the transmission of social norms influencing these activity specific behaviors could prove particularly relevant. Studies with longer follow-up would also be useful for a better assessment of the long-term combined impact of unhealthy eating habits and high TV exposure among boys.

## Supplementary information

**Additional file 1.** Additional Table 1: Bayesian Information Criterion (BIC) value and number of relevant variables by number of clusters at ages 2 and 5. Additional Table 2: Probability multinomial distribution of relevant variables by clusters among boys at 2 years of age. Additional Table 3: Probability multinomial and Gaussian distribution of relevant variables by cluster among girls at 2 years of age. Additional Table 4: Probability multinomial and Gaussian distribution of relevant variables by cluster among boys at 5 years of age. Additional Table 5: Probability multinomial and Gaussian distribution of relevant variables by cluster among girls at 5 years of age.

## Data Availability

All data supporting the findings of this study are included in the present article or the supplemental material.

## Abbreviations

BIC: Bayesian information criterion
EDNP: Energy-dense nutrient-poor
OW-OB: Overweight and obesity
PA: Physical activity
PCA: Principal component analysis
SEP: Socio-economic position
