# Supplementary material for: Clusters of diet, physical activity, television exposure and sleep habits and their association with adiposity in preschool children: the EDEN mother-child cohort
Source: Int J Behav Nutr Phys Act. 2020 Feb 12;17:20. doi: 10.1186/s12966-020-00927-6 (PMC7014717; doi:10.1186/s12966-020-00927-6)
Supplement: Supplementary file 1 — Additional file 1. Additional Table 1: Bayesian Information Criterion (BIC) value and number of relevant variables by number of clusters at ages 2 and 5. Additional Table 2: Probability multinomial distribution of relevant variables by clusters among boys at 2 years of age. Additional Table 3: Probability multinomial and Gaussian distribution of relevant variables by cluster among girls at 2 years of age. Additional Table 4: Probability multinomial and Gaussian distribution of relevant variables by cluster among boys at 5 years of age. Additional Table 5: Probability multinomial and Gaussian distribution of relevant variables by cluster among girls at 5 years of age. [file 12966_2020_927_MOESM1_ESM.docx]

Additional Table 1: Bayesian Information Criterion (BIC) value and number of relevant variables by number of clusters at ages 2 and 5.

| Numbers of clusters | At 2 years of age | | | | At 5 years of age | | | |
| --- | --- | --- | --- | --- | --- | --- | --- | --- |
|  | BOYS | | GIRLS | | BOYS | | GIRLS | |
|  | BIC value | Relevant variables | BIC value | Relevant variables | BIC value | Relevant variables | BIC value | Relevant variables |
| 1 | -40275 | 44/44 | -36885 | 44/44 | -32507 | 40/40 | -28234 | 40/40 |
| 2 | **-39323** | **15/44** | **-36240** | **17/44** | **-32042** | **14/40** | -27800 | 15/40 |
| 3 | -39355 | 14/44 | -36301 | 9/44 | -32054 | 9/40 | -27801 | 6/40 |
| 4 | -39516 | 3/44 | -36349 | 3/44 | -32077 | 6/40 | **-27791** | **5/40** |
| 5 | -39539 | 3/44 | -36384 | 2/44 | -32088 | 6/40 | -27808 | 4/40 |
| 6 | -39566 | 3/44 | -36420 | 1/44 | -32117 | 5/40 | -27824 | 4/40 |
| 7 | -39585 | 3/44 | -36430 | 1/44 | -32141 | 3/40 | -27839 | 4/40 |

Numbers in bold correspond to the lowest BIC value, indicative of the best model.

Additional Table 2: Probability multinomial distribution of relevant variables by clusters among boys at 2 years of age.

| CLUSTER LABELS | | ‘UNHEALTHY EATING’ (N=340) | ‘HEALTHY EATING’ (N=410) |
| --- | --- | --- | --- |
| *SWEETENED BEVERAGES* | |  |  |
| Soft drinks | Never | 0.16 | **0.63** |
|  | <1 time/month | 0.11 | 0.17 |
|  | 1–3 times/month | 0.23 | 0.09 |
|  | 1–3 times/week | **0.23** | 0.06 |
|  | 4–6 times/week | 0.12 | 0.02 |
|  | 1 time/day | 0.10 | 0.02 |
|  | Several times/day | 0.06 | 0.01 |
| Sweetened beverages at meals, Yes/No | | **0.34/**0.66 | 0.09/**0.91** |
| Fruit juice | Never | 0.04 | **0.21** |
|  | <1 time/month | 0.06 | 0.16 |
|  | 1–3 times/month | 0.17 | 0.21 |
|  | 1–3 times/week | **0.32** | 0.20 |
|  | 4–6 times/week | 0.14 | 0.09 |
|  | 1 time/day | 0.22 | 0.12 |
|  | Several times/day | 0.04 | 0.01 |
| Diet soft drinks | Never | **0.71** | **0.93** |
|  | <1 time/month | 0.09 | 0.04 |
|  | 1–3 times/month | 0.09 | 0.01 |
|  | 1–3 times/week | 0.06 | 0.02 |
|  | 4–6 times/week | 0.03 | 0.00 |
|  | 1 time/day | 0.01 | 0.00 |
|  | Several times/day | 0.01 | 0.00 |
| *PROCESSED AND FAST FOOD* | |  |  |
| Processed meat | Never | 0.07 | **0.42** |
|  | <1 time/month | 0.12 | 0.28 |
|  | 1–3 times/month | **0.41** | 0.21 |
|  | 1–3 times/week | 0.32 | 0.07 |
|  | 4–6 times/week | 0.05 | 0.02 |
|  | 1 time/day | 0.03 | 0.00 |
| Pizza/pie | Never | 0.06 | **0.36** |
|  | <1 time/month | 0.20 | 0.35 |
|  | 1–3 times/month | **0.53** | 0.26 |
|  | 1–3 times/week | 0.21 | 0.03 |
|  | 4–6 times/week | 0.00 | 0.01 |
| French fries/chips | Never | 0.00 | 0.16 |
|  | <1 time/month | 0.06 | 0.30 |
|  | 1–3 times/month | **0.50** | **0.45** |
|  | 1–3 times/week | 0.41 | 0.08 |
|  | 4–6 times/week | 0.03 | 0.01 |
|  | 1 time/day | 0.00 | 0.00 |
|  | Several times/day | 0.00 | 0.00 |
| Potato chips/crisps | Never | 0.02 | 0.19 |
|  | <1 time/month | 0.15 | 0.35 |
|  | 1–3 times/month | **0.49** | **0.37** |
|  | 1–3 times/week | 0.30 | 0.08 |
|  | 4–6 times/week | 0.03 | 0.01 |
|  | 1 time/day | 0.01 | 0.00 |
|  | Several times/day | 0.00 | 0.00 |
| *FRUITS AND VEGETABLES* | |  |  |
| Cooked vegetables | Never | 0.01 | 0.01 |
|  | <1 time/month | 0.03 | 0.01 |
|  | 1–3 times/month | 0.10 | 0.05 |
|  | 1–3 times/week | **0.32** | 0.15 |
|  | 4–6 times/week | 0.29 | 0.25 |
|  | 1 time/day | 0.24 | **0.33** |
|  | Several times/day | 0.01 | 0.20 |
| Legumes | Never | 0.06 | 0.25 |
|  | <1 time/month | 0.24 | 0.27 |
|  | 1–3 times/month | **0.49** | **0.36** |
|  | 1–3 times/week | 0.19 | 0.10 |
|  | 4–6 times/week | 0.01 | 0.02 |
|  | 1 time/day | 0.01 | 0.00 |
|  | Several times/day | 0.00 | 0.00 |
| Stewed fruit | Never | 0.02 | 0.01 |
|  | <1 time/month | 0.03 | 0.03 |
|  | 1–3 times/month | 0.07 | 0.07 |
|  | 1–3 times/week | **0.33** | 0.15 |
|  | 4–6 times/week | 0.25 | 0.26 |
|  | 1 time/day | 0.28 | **0.37** |
|  | Several times/day | 0.02 | 0.11 |
| *NON-CORE SWEETS* | |  |  |
| Chocolate/candy | Never | 0.01 | 0.18 |
|  | <1 time/month | 0.04 | 0.13 |
|  | 1–3 times/month | 0.18 | 0.23 |
|  | 1–3 times/week | **0.33** | **0.28** |
|  | 4–6 times/week | 0.22 | 0.10 |
|  | 1 time/day | 0.19 | 0.07 |
|  | Several times/day | 0.03 | 0.01 |
| Dairy pudding/ice cream | Never | 0.02 | 0.14 |
|  | <1 time/month | 0.07 | 0.18 |
|  | 1–3 times/month | 0.24 | **0.23** |
|  | 1–3 times/week | **0.30** | 0.19 |
|  | 4–6 times/week | 0.17 | 0.12 |
|  | 1 time/day | 0.13 | 0.11 |
|  | Several times/day | 0.07 | 0.03 |
| Cookies/biscuits | Never | 0.00 | 0.04 |
|  | <1 time/month | 0.00 | 0.03 |
|  | 1–3 times/month | 0.03 | 0.08 |
|  | 1–3 times/week | 0.17 | 0.24 |
|  | 4–6 times/week | 0.21 | 0.21 |
|  | 1 time/day | **0.44** | **0.37** |
|  | Several times/day | 0.15 | 0.03 |
| *BABY FOODS consumers*, Yes/No | | 0.49/**0.51** | **0.72/**0.28 |

Numbers in bold are the mode value.

Additional Table 3: Probability multinomial and Gaussian distribution of relevant variables by cluster among girls at 2 years of age.

| CLUSTER LABELS | | ‘UNHEALTHY EATING’ (N=298) | ‘HEALTHY EATING’ (N=388) |
| --- | --- | --- | --- |
| *SWEETENED BEVERAGES* | |  |  |
| Soft drinks | Never | 0.22 | **0.75** |
|  | <1 time/month | 0.14 | 0.13 |
|  | 1–3 times/month | **0.23** | 0.09 |
|  | 1–3 times/week | 0.21 | 0.03 |
|  | 4–6 times/week | 0.06 | 0.00 |
|  | 1 time/day | 0.09 | 0.00 |
|  | Several times/day | 0.05 | 0.00 |
| Fruit juice | Never | 0.07 | **0.25** |
|  | <1 time/month | 0.05 | 0.17 |
|  | 1–3 times/month | 0.18 | 0.21 |
|  | 1–3 times/week | **0.31** | 0.20 |
|  | 4–6 times/week | 0.15 | 0.06 |
|  | 1 time/day | 0.20 | 0.10 |
|  | Several times/day | 0.04 | 0.01 |
| Diet soft drinks | Never | **0.69** | **0.92** |
|  | <1 time/month | 0.07 | 0.06 |
|  | 1–3 times/month | 0.11 | 0.02 |
|  | 1–3 times/week | 0.08 | 0.00 |
|  | 4–6 times/week | 0.03 | 0.00 |
|  | 1 time/day | 0.02 | 0.00 |
| Sweetened beverages at meals, Yes/No | | 0.22/**0.78** | 0.05/**0.95** |
| *PROCESSED AND FAST FOOD* | |  |  |
| Potato chips/crisps | Never | 0.04 | 0.20 |
|  | <1 time/month | 0.08 | **0.37** |
|  | 1–3 times/month | **0.51** | 0.35 |
|  | 1–3 times/week | 0.31 | 0.07 |
|  | 4–6 times/week | 0.05 | 0.01 |
|  | 1 time/day | 0.01 | 0.00 |
| French fries/chips | Never | 0.01 | 0.15 |
|  | <1 time/month | 0.10 | 0.27 |
|  | 1–3 times/month | 0.41 | **0.47** |
|  | 1–3 times/week | **0.44** | 0.11 |
|  | 4–6 times/week | 0.03 | 0.00 |
|  | 1 time/day | 0.01 | 0.00 |
| Processed meat | Never | 0.11 | **0.35** |
|  | <1 time/month | 0.15 | 0.31 |
|  | 1–3 times/month | **0.33** | 0.26 |
|  | 1–3 times/week | 0.31 | 0.06 |
|  | 4–6 times/week | 0.07 | 0.02 |
|  | 1 time/day | 0.03 | 0.00 |
| Pizza/pie | Never | 0.12 | **0.29** |
|  | <1 time/month | 0.19 | 0.35 |
|  | 1–3 times/month | **0.50** | 0.29 |
|  | 1–3 times/week | 0.18 | 0.06 |
|  | 4–6 times/week | 0.01 | 0.01 |
|  | 1 time/day | 0.00 | 0.00 |
| *BABY FOODS consumers,* Yes/No | | **0.52/**0.48 | **0.73/**0.27 |
| *NON-CORE SWEETS* | |  |  |
| Chocolate/candy | Never | 0.02 | 0.11 |
|  | <1 time/month | 0.02 | 0.16 |
|  | 1–3 times/month | 0.16 | 0.26 |
|  | 1–3 times/week | **0.32** | **0.31** |
|  | 4–6 times/week | 0.25 | 0.08 |
|  | 1 time/day | 0.19 | 0.08 |
|  | Several times/day | 0.04 | 0.00 |
| Cookies/biscuits | Never | 0.00 | 0.00 |
|  | <1 time/month | 0.00 | 0.05 |
|  | 1–3 times/month | 0.05 | 0.13 |
|  | 1–3 times/week | 0.16 | **0.33** |
|  | 4–6 times/week | 0.25 | 0.23 |
|  | 1 time/day | **0.40** | 0.25 |
|  | Several times/day | 0.14 | 0.01 |
| *VEGETABLES* | |  |  |
| Cooked vegetables | Never | 0.02 | 0.00 |
|  | <1 time/month | 0.01 | 0.01 |
|  | 1–3 times/month | 0.09 | 0.05 |
|  | 1–3 times/week | **0.32** | 0.15 |
|  | 4–6 times/week | 0.26 | 0.26 |
|  | 1 time/day | 0.26 | **0.32** |
|  | Several times/day | 0.04 | 0.21 |
| TV EXPOSURE | |  |  |
| TV/DVD watching time | 0 min/day | 0.04 | 0.18 |
|  | >0 to ≤30 min/day | **0.38** | **0.49** |
|  | >30 to ≤60 min/day | 0.27 | 0.17 |
|  | >60 min/day | 0.31 | 0.16 |
| TV on during meals | Never | **0.33** | **0.48** |
|  | Sometimes | 0.26 | 0.31 |
|  | Often | 0.24 | 0.15 |
|  | Always | 0.17 | 0.06 |
| PHYSICAL ACTIVITY | |  |  |
| Outdoor play time (standardized by season) min/day, mean (SD) | | 3 (60) | -13 (51) |
| SLEEP |  |  |  |
| Regular wake-up time, Yes/No | | **0.84/**0.16 | **0.93/**0.07 |
| Regular bedtime, Yes/No | | **0.90/**0.10 | **0.99/**0.01 |

Numbers in bold are the mode value. Abbreviations: min minute, SD standard deviation.

Additional Table 4: Probability multinomial and Gaussian distribution of relevant variables by cluster among boys at 5 years of age.

| CLUSTER LABELS | | ‘HIGH TV - UNHEALTHY  EATING’ (N=325) | ‘MODERATE TV - HEALTHY EATING’ (N=308) |
| --- | --- | --- | --- |
| TV EXPOSURE | |  |  |
| TV on during meals | Never | 0.18 | **0.55** |
|  | Sometimes | **0.33** | 0.32 |
|  | Often | 0.32 | 0.10 |
|  | Always | 0.18 | 0.03 |
| TV/DVD watching time, min/day, mean (SD) | | 107 (49) | 58 (29) |
| *SWEETENED BEVERAGES* | |  |  |
| Sweetened beverages at meals, Yes/No | | 0.35/**0.65** | 0.04/**0.96** |
| Soft drinks | Never | 0.06 | 0.16 |
|  | <1 time/month | 0.05 | 0.26 |
|  | 1–3 times/month | 0.19 | **0.30** |
|  | 1–3 times/week | **0.30** | 0.21 |
|  | 4–6 times/week | 0.15 | 0.04 |
|  | 1 time/day | 0.16 | 0.02 |
|  | Several times/day | 0.08 | 0.00 |
| Fruit juice | Never | 0.03 | 0.05 |
|  | <1 time/month | 0.01 | 0.10 |
|  | 1–3 times/month | 0.12 | 0.22 |
|  | 1–3 times/week | 0.28 | 0.20 |
|  | 4–6 times/week | 0.17 | 0.11 |
|  | 1 time/day | **0.29** | **0.31** |
|  | Several times/day | 0.09 | 0.02 |
| *PROCESSED AND FAST FOOD* | |  |  |
| French fries/chips | Never | 0.00 | 0.01 |
|  | <1 time/month | 0.07 | 0.17 |
|  | 1–3 times/month | 0.40 | **0.64** |
|  | 1–3 times/week | **0.49** | 0.19 |
|  | 4–6 times/week | 0.02 | 0.00 |
|  | 1 time/day | 0.01 | 0.00 |
| Potato chips/crisps | Never | 0.03 | 0.05 |
|  | <1 time/month | 0.14 | 0.28 |
|  | 1–3 times/month | **0.45** | **0.56** |
|  | 1–3 times/week | 0.37 | 0.12 |
|  | 4–6 times/week | 0.02 | 0.00 |
|  | 1 time/day | 0.00 | 0.00 |
| Processed meat | Never | 0.07 | 0.06 |
|  | <1 time/month | 0.09 | 0.23 |
|  | 1–3 times/month | 0.36 | **0.47** |
|  | 1–3 times/week | **0.39** | 0.23 |
|  | 4–6 times/week | 0.06 | 0.01 |
|  | 1 time/day | 0.02 | 0.00 |
|  | Several times/day | 0.01 | 0.00 |
| *SNACKING* | 2 times/day or more | 0.05 | 0.01 |
|  | 1 time/day | 0.23 | 0.05 |
|  | Every 2-3 days | 0.24 | 0.06 |
|  | Less often | **0.39** | **0.51** |
|  | Never | 0.10 | 0.38 |
| *NON-CORE SWEETS* | |  |  |
| Chocolate/candy | Never | 0.00 | 0.03 |
|  | <1 time/month | 0.01 | 0.07 |
|  | 1–3 times/month | 0.12 | 0.27 |
|  | 1–3 times/week | **0.36** | **0.45** |
|  | 4–6 times/week | 0.24 | 0.10 |
|  | 1 time/day | 0.24 | 0.00 |
|  | Several times/day | 0.03 | 0.01 |
| Cookies/biscuits | Never | 0.01 | 0.01 |
|  | <1 time/month | 0.00 | 0.05 |
|  | 1–3 times/month | 0.05 | 0.12 |
|  | 1–3 times/week | 0.16 | **0.31** |
|  | 4–6 times/week | 0.23 | 0.23 |
|  | 1 time/day | **0.42** | 0.27 |
|  | Several times/day | 0.13 | 0.01 |
| PHYSICAL ACTIVITY | |  |  |
| Organized sportive activity, Yes/No | | 0.41/**0.59** | **0.64/**0.36 |
| Walking time, min/day, mean (SD) | | 50 (49) | 36 (29) |
| SLEEP |  |  |  |
| Sleep duration, hours/day, mean (SD) | | 10h46 (0h30) | 10h58 (0h27) |

Numbers in bold are the mode value. Abbreviations: min minute, SD standard deviation.

Additional Table 5: Probability multinomial and Gaussian distribution of relevant variables by cluster among girls at 5 years of age.

| CLUSTER LABELS | | | ‘Low TV–low outdoor PA’  (N=171) | | ‘Moderate TV–rather high outdoor PA’ (N=111) | | ‘High TV-low outdoor PA’  (N=242) | | ‘Very high TV-high outdoor PA’  (N=38) |
| --- | --- | --- | --- | --- | --- | --- | --- | --- | --- |
| TV EXPOSURE | | |  | |  | |  | |  |
| TV/DVD watching time |  | | |  | | | |  | |
| min/day, mean (SD) | | | 35 (15) | | 62 (29) | | 91 (34) | | 174 (45) |
| TV on during meals | | |  | |  | |  | |  |
| Never | | | **0.71** | | 0.34 | | 0.21 | | 0.07 |
| Sometimes | | | 0.29 | | **0.49** | | 0.32 | | 0.22 |
| Often | | | 0.00 | | 0.11 | | **0.35** | | 0.30 |
| Always | | | 0.00 | | 0.06 | | 0.11 | | **0.41** |
| PHYSICAL ACTIVITY | | | | |  | |  | |  |
| Walking time | | |  | |  | |  | |  |
| min/day, mean (SD) | | | 30 (16) | | 57 (27) | | 34 (16) | | 73 (49) |
| Outdoor play time**,** standardized by season | | | |  | | | | | |
| min/day, mean (SD) | | | -32 (31) | | 34 (62) | | -24 (38) | | 32 (78) |
| DIET | | |  | |  | |  | |  |
| Sweetened beverages at meals | |  | | | |  | | | |
| Yes/No | | | 0.02/**0.98** | | 0.21/**0.79** | | 0.19/**0.81** | | 0.39/**0.61** |

Numbers in bold are the mode value. Abbreviations: min minute, SD standard deviation.
